# Canopy-cooling systems applied on avocado trees to mitigate heatwaves damages

**DOI:** 10.1038/s41598-022-16839-3

**Published:** 2022-07-22

**Authors:** Silit Lazare, Helena Vitoshkin, Victor Alchanatis, Guy Reshef, Dafna Ziv, Eli Simenski, Arnon Dag

**Affiliations:** 1 Gilat Research Center, Agricultural Research Organization, Volcani Institute, M.P. Negev 8528000, Gilat, Israel; 2 Institute of Agricultural Engineering, Agricultural Research Organization, Volcani Institute, Rishon LeTsiyon 7505101, Israel; 3 Netafim Ltd, Hatzerim, Israel; 4 The Extension Service, Ministry of Agriculture and Rural Development, Beit Dagan, Israel; 5 NevaTeam, Avigdor, Israel

**Keywords:** Plant sciences, Climate sciences, Natural hazards

## Abstract

With climate change, spring heatwaves have become frequent in the Mediterranean region. High temperatures combined with wind and low humidity are problematic for subtropical crops adapted to high humidity and mild climate. Avocado is a valuable crop—nutritionally and economically—and many new orchards are planted in Mediterranean areas. Spring heatwaves increase avocado fruitlets dropping, severely decreasing yields. Addressing and solving the problem are necessary to maintain the crop's profitability. This study presents a sprinkler-based canopy cooling method that uses the existing pressurized irrigation system. The study aimed to test the system's performance during spring heatwaves, after the flowering season, in avocado orchards cultivated in a semi-arid region. The experiments examined the effect of various sprinkler types with varying flow rates and installation methods: sprayers, sprinklers and pulsing sprinklers, on foliage temperature, stem water potential, salt accumulation in the leaf, fruitlet survival and yield. The system reduced leaf temperatures by approximately 10 °C, significantly decreasing the trees' drought stress and increasing yields by 8–12%. Using low-quality water is possible, but requires adjustments to avoid salt damage to the leaves. The system can mitigate heat stress, and provides a relatively simple solution for handling spring heatwaves. The evaporative cooling system is modeled for semi-desert and desert conditions; the dry, windy climate contributes to the method's effectiveness.

## Introduction

Climate change is one of the modern threats to agriculture and food security. The air temperature on earth has been constantly increasing during the last decades^1^; according to the intergovernmental climate change panel (ICCP), global warming of 1.5 °C is predicted in the next 10–30 years^2^. The Mediterranean region has been identified as one of the world's most vulnerable and susceptible regions to climate change^3^. In this region, besides general warming, spring heatwaves from the desert have become common^4,5^. Heatwaves are natural hazards of extremely high temperatures, affecting various life sectors, including agriculture^6^, and farmers need to cope with obstacles to growth and yield loss. Heatwaves cannot be characterized by specific thresholds; rather, they are a region-relative combination of high temperatures and low air humidity^7^.

Avocado (*Persea americana*) is a crop with high nutritional and economic value, and its cultivation areas have expanded quickly^8^. The Mediterranean region is one of the areas where avocado became a major crop^9^. Avocado suffers from high susceptibility to abiotic stress stemming from environmental conditions, e.g., drought^10^, salinity^11^, frost^12^, heat^13^ and others. Spring heatwaves can damage both the vegetative growth of avocado and its reproductive development; during this season the fruitlets are young and tend to be abscised in response to stress^14,15^. Regarding Hass, half of the interannual variance in yield was explained by heat stress^16^. Several studies have suggested that climate change will have a direct and indirect effect on avocado in general and on the Hass cultivar specifically^17–19^. Climate change will likely cause phenological changes through increased temperature, decreased precipitation, decreased water infiltration and higher intensity and duration of climatic events, which will eventually lead to a reduction in productivity^20^. The climate in indigenous habitats suggests that avocado will be intolerant of extreme heat. Such heat could also be more detrimental during critical periods such as pollination and fruit set. During spring, when the avocado flowers and fruit sets, hot dry winds, known as Santa Ana winds in California, Sharav or Hamsin in Israel and Berg winds in South Africa, can considerably reduce yields^21^.

Several methods were previously suggested to help fruit trees cope with heat stress, among them using heat-tolerant rootstocks^22^, net shading^23,24^, particle film application^25^, fertilization manipulation^26^, and supplementary irrigation^27^. Most of these methods are time-consuming, and/or entail additional expenses. The unpredictable nature of heatwaves requires an efficient, low-cost method, which should be available for immediate use, responsive and easy to implement. Supplementary irrigation is very important; plants under water stress are likely to suffer from heat stress as well^28^. Supplemental irrigation is probably the easiest method to implement because it uses the existing system. However, under high vapor pressure deficit (VPD), the tree closes its stomata, reducing the transpiration rate^29^. Consequently, water transport is not optimal, because it relies primarily on root pressure^30^.

The above-canopy evaporative cooling method has been studied for several decades as a potential solution for crop protection against drought and heat stress^31–34^. This method explores sprinkling operated above the crop canopy, which effectively reduces air and leaf temperatures through latent heat transfer, thereby reducing VPD^35^. The method is especially effective in hot and dry climates. Therefore, short-term treatment can reduce plant damage during extreme heatwaves. However, adapting the method to a specific crop at a specific developmental stage, under specific climatic conditions is not straightforward. Published experiments on different types of crops in several growth locations yielded a wide variation of results. Although sprinkling might positively affect one kind of crop^34^, it has no significant effect on another^36^. Fluctuations of air humidity and temperatures determine the efficiency of the cooling methods; e.g., Evans^32^ shows that a sprinkling, pulsing flow rate of approximately 3L/s/ha is sufficient to achieve fruit cooling. However, a doubled flow rate in short pulsing intervals is required in high wind or higher ambient temperature. Droplet size and sprinkler spacing were also found to affect the uniformity of the application's results^37^. Furthermore, the increased use of low-quality water for irrigation due to a shortage of freshwater creates a potential hazard of salt accumulation in the target plant's leaves, especially in salt-sensitive crops like avocado^38^. The effectiveness of evaporative cooling above trees has barely been studied in subtropical crops, such as avocado and mango. Moreover, most of the previous tree-related work focused on the effect of heat stress on the fruit quality, while the present work examines fruitlet survival, i.e., the impact during an early developmental stage, which significantly affects the trees' productivity and final fruit yields.

The work presented evaluates the use of the irrigation system for overhead canopy cooling in avocado orchards. The effect of several possible sprinkling methods and two different levels of water quality on the stress response of trees during a severe heatwave, and on their final yields, are examined. The results of this study can contribute to the global efforts to cope with climate change challenges in horticulture, specifically fruticulture.

## Materials and methods

The experiments were conducted in two 'Hass' avocado orchards, located near Kibbutz Gevim (31°30′27″ N 34°35′55″ E) and near Kibbutz Sa'ad (31°28′13″ N 34°32′6″ E), both in the Western Negev region of Israel. The soils of this area are Calcic Xerosols with a texture of sandy loam^39^.

**Figure 1:**
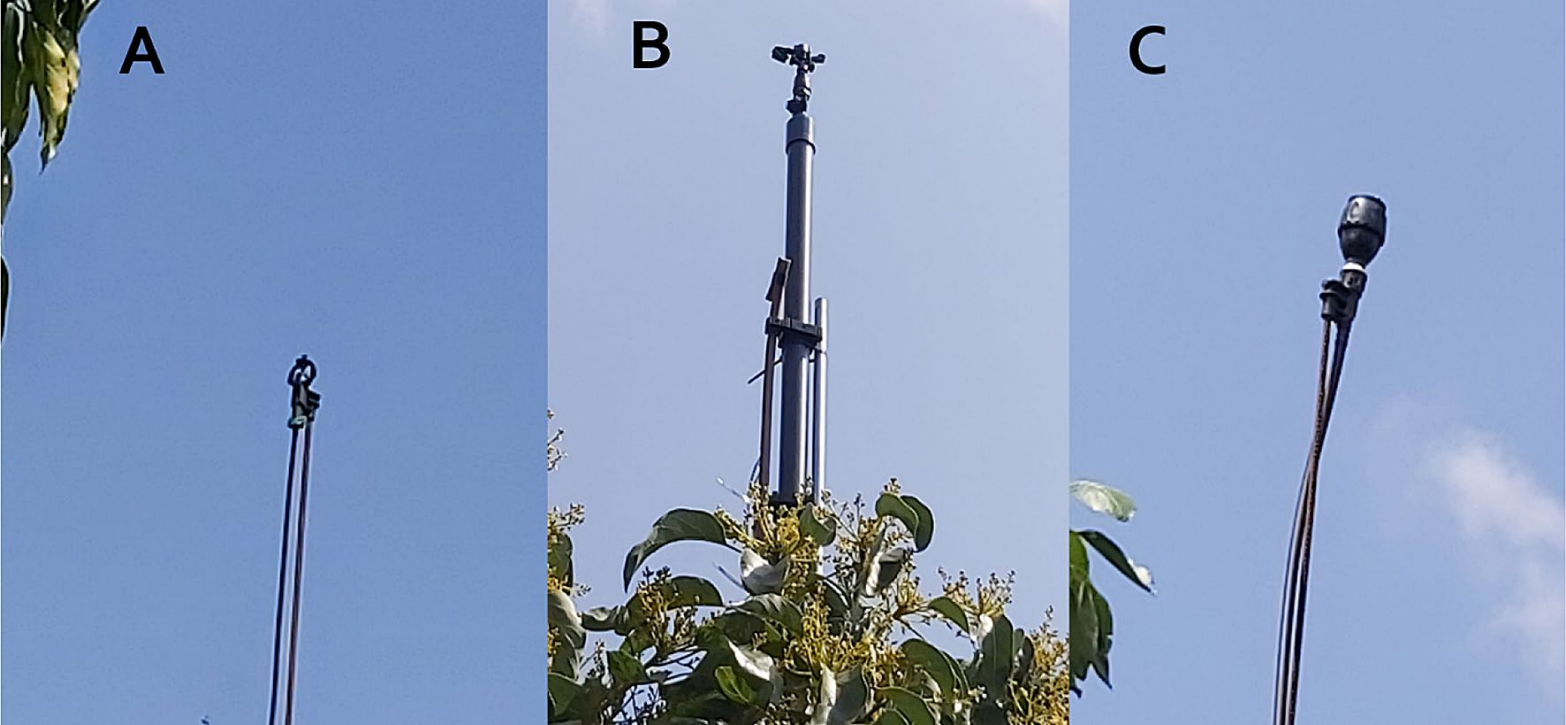
The sprinklers usesd for evaporation cooling methos. (**A**) Mini sprinkler- 'Super-Net', Netafim, one per tree, 50 L/h', irrigation rate—21 m^3^/h/ha. (**B**) Sprinkler in pulses 'D-Net', Netafim, every second tree, 370 L/h, activated in pulses—on/off 10/10 s, irrigation rate of 17 m^3^/h/ha). (**C**). Sprinkler—'Mega-Net', Netafim, every second tree, 450 L/h, irrigation rate of 31 m^3^/h/ha).

The Gevim orchard consists of 27 rows, each with 33 yielding trees, of which 790 were 'Hass' and 97 were 'Ettinger'; the latter served as the pollenizer. The trees were planted in 2009, at a density of 6 × 4 m (420 trees per ha). At the time of the study, the trees' height was approximately 3 m and their width approximately 3 m. The trees were drip irrigated, using emitters with a flow rate of 1.1 L/h spaced every 0.3 m in two lateral lines per tree row. The amount of water per tree per month varied during the year (here we describe only the experiment months): April—1.4 m^3^, May—3.1, June—3.6, July—3.9, August—3.9, September—3.5, October—2.4 and November—1.1. The fruit was harvested on 8.11.2020. Three types of canopy cooling systems were examined: sprayers (Super-Net, Netafim, 50 L/h, one per tree, irrigation rate of 21 m^3^/h/ha); sprinklers (Mega-Net, Netafim, 450 L/h, 12 × 12 m placement, irrigation rate of 31 m^3^/h/ha); and sprinklers in pulses (D-Net, Netafim, 370 L/h, 12 × 12 m, activated in pulses—on/off 10/10 s, irrigation rate of 17 m^3^/h/ha) (Fig. 1). Treatments differ one from each other in droplet size, water application radius, continues vs. pulses operation and irrigation rate as mentioned above. All the sprinklers were located approximately 0.5 m above the canopy, i.e., 3.5 m above the ground. Two plots were allocated for each type of cooling system, with a total of six treated plots, and two untreated control plots. At the center of each plot, three similar trees, surrounded by the same treatment trees, were marked for specific measurements during the season. All plots were inside the orchard and surrounded by other trees. The cooling systems were based on the brackish water (BW) that is used for irrigation. Water analysis revealed 125.8 ppm of Na, 183.0 ppm of Cl, and electrical conductivity (EC) of 1.10 ds/m.

The Sa'ad orchard consisted of 48 rows, each with 14 yielding trees. Here as well, the 'Hass' avocado was the examined cultivar, with 'Ettinger' as the pollenizer. The trees were planted in 2009, at a density of 7 × 5 m (410 trees per ha). At the time of the study, the trees were approximately 7 m tall and approximately 4 m wide. The trees were drip irrigated using emitters with a flow rate of 1 L/h spaced every 0.3 m in two lateral lines per tree row. Each tree was irrigated daily. The water per tree per month varied throughout the year (water quantity in m^3^): April 2.3, May 2.9, June 3.3, July 3.7, August 3.6, September 3.6, October 3.3, November 1.6 and December 1.3. The fruit was harvested on December 28, 2020. Two types of cooling system were tested: sprayers (Super-Net Netafim 50 L/h, one per tree, irrigation rate of 21 m^3^/h/ha); and sprinklers (Mega-Net, Netafim, 450 L/h, 10.5 × 14 m placement, irrigation rate of 31 m^3^/h/ha). All the sprinklers were located approximately 0.5 m above the canopy, i.e., 7.5 m above the ground. Two plots were assigned to each treatment and two untreated plots were used for control. All the treatment plots were in the same row. Another control plot was located in a different row nearby. At the center of each plot, three similar trees, surrounded by the same treatment trees, were marked for specific measurements during the season. The cooling systems were based on fresh water (FW), but the trees were irrigated with the same BW used in Gevim. Fresh water analysis revealed 22.1 ppm of Na, 23.0 ppm of Cl and EC of 0.38 ds/m.

At both sites, the thresholds for automated activation of the cooling system were set to air temperature above 33 °C or humidity below 40%. Flowering at both sites began during the first week of April, 2020 and continued for 3–4 weeks. Bee hives were present during the flowering season and were removed from the orchards during the first week of May. The cooling systems were first activated on May 16, 2020, at the beginning of an extreme heatwave that lasted six days. All automatic activation events are presented in Table 1. In addition, two preventative salt washing events were carried out manually in the Gevim orchard, on May 23^rd^ and June 8th, to prevent salt-induced damage to leaves. They each lasted three hours at 16–18 °C temperatures and 100% humidity, toward the end of the night. Starting on June 12, 2020, only the FW system was activated, to avoid exposing the trees to more saline.

**Table 1:** Orchards' microclimate characteristics, and the days and hours the cooling system was applied.

Date	Ambient conditions when system activated		Operating hours, BW (Gevim)	Operating hours, FW (Sa'ad)
	Air temperature, °C	Relative humidity, %		
May 16, 2020	39	20	10:00–18:00	10:00–18:00
May 17, 2020	44	50	10:00–19:00	10:00–19:30
May 18, 2020	41	65	9:00–20:50	9:00–16:45
May 19,2020	41	50	8:00–22:00	8:30–19:00
May 20, 2020	40	50	9:00–21:30	9:00–19:00
May 21, 2020	40	50	9:00–19:00	9:40–18:00
May 23, 200	18	100	3:00–6:00	–-
June 7, 2020	32	35	12:00–13:00	12:00–13:00
June 8, 2020	16	100	3:00–6:00	–-
June 9, 2020	33	24	–-	9:30–15:30
June 17, 2020	36	19	–-	11:42–17:00
June 21, 2020	32	31	–-	12:30–14:00

### Measurements

#### Weather conditions

Ambient weather conditions were measured at standard meteorological stations, one approximately 4 km away from Gevim, and the other about 1 km away from Sa'ad. Relevant temperature and humidity data are given in Table 1. During all the experiments, air temperatures were higher than 32 °C, reaching a maximum of 44 °C; and the relative humidity was lower than 65%, reaching a minimum of 19%. During all the cooling periods, the wind speed was in the range of 2–9 m/s, primarily from the East.

#### Canopy temperature

A data sampling and control system was installed in each orchard to collect leaf temperature data, measured with T-type thermocouples attached to the inside of the leaves, i.e., the sensors were under the shade of a leaf to avoid direct solar radiation. After pre-calibration, the thermocouples were installed on two trees under the sprinkler treatment and on two control trees in each plot, in both orchards. To investigate the effect of evaporative cooling on foliage temperature at different levels of a tree, six thermocouples were randomly spread in the same trees at heights of 1 m and 2.5–3 m. Some of measurements were approximate; strong winds blew some of the thermocouples from the leaves. In those cases, the air temperature of the leaf's boundary layer was measured. In this study, we assumed that the air temperature in the vicinity of a leaf is very close to the leaf's temperature. In addition, the canopy temperature of the trees at Gevim was mapped on May 19, 2020, during the major heatwave, using a thermal infrared camera (SC655 from FLIR) mounted on a drone. The camera is radiometrically calibrated, based on an uncooled microbolometer focal array, with a sensitivity of 0.1 degrees and a radiometric accuracy of less than 2 degrees. The images were acquired from 50 m above ground level (AGL) with a 24 mm lens, resulting in a ground spatial resolution of 3 cm/pixel. The flight plan included 70% overlap between adjacent legs, and 90% overlap in the flight direction. Seven ground control points were placed within the scanned area, and their geographical coordinates were measured using an RTK GPS, with 1 cm accuracy. The map produced was georeferenced from the acquired images using Pix4d commercial software.

#### Salt leaf concentration

Twenty mature healthy leaves from the canopy of two trees per plot were sampled on May 27, 2020, a week after the major heatwave ended. The leaves were dried at 70 °C in a well-ventilated oven. Then, each sample was ground and thoroughly mixed. The quantity of Cl in the leaf was determined based on water extraction (0.1 g dry matter in 10 mL deionized water), using an MKII Sherwood M926 Chloride Analyzer. Na and Ca were determined by digesting the powdered material with nitric acid and H_2_O_2_ and analyzed using ICP-OES 5100 (Agilent Technologies). In addition, the orchard was mapped from a drone, using an RGB camera (SONY ILCE-600); the procedure that was used with the thermal camera, described above, was followed.

#### Stem water potential (SWP)

On May 18, 2020, the third day of the heatwave, two mature leaves from each of four trees per plot were enclosed in aluminum bags for two hours before measuring. SWP was measured during noon hours using a Scholander-type pressure chamber (MRC, Israel).

#### Fruitlet survival survey

At the center of each treatment plot, three similar trees were marked for surveys of fruitlets. Twenty uniform inflorescences per tree had been marked before the experiment began, and the number of fruitlets per inflorescence were counted on May 13, 2020, May 27, 2020, and August 10, 2020.

#### Yield

The fruit was harvested on November 11, 2020, in Gevim and on December 28, 2020, in Sa'ad, four trees per plot. Each tree was harvested individually, and its yield weighed. Fifty random fruits were counted and weighed separately to calculate the average weight of a single fruit and the number of fruits per tree. To calculate the total yield per ha, we multiplied the average yield of a single tree by the number of trees per ha, i.e.—420 in Gevim or 410 in Sa'ad.

### Statistical analysis

JMP®14.0.0 software (SAS Institute Inc.) was used to carry out ANOVA. Tukey–Kramer test was used to estimate the differences between the treatments (SWP, Na, Cl and Ca in leaves, Fruiulets per inflorescence, and yield indices). The effect of cooling treatment on canopy temperature (thermal imaging) was tested using the following procedure: The tree's canopy was delineated using GIS platform (QGIS 3.26). For each plot, nine trees were marked, resulting in 18 marked trees per treatment. For each tree, a polygon was manually created on the thermal image. Then, the 'zonal statistics tool was used (QGIS toolbox) to calculate the mean temperature of each tree. A single factor ANOVA test with the Tukey- Kramer was used for comparing between canopy temperature of the different treatments.

### Ethical approval

All experiments related to plants were conducted in accordance with the relevant guidelines.

## Results

**Figure 2:**
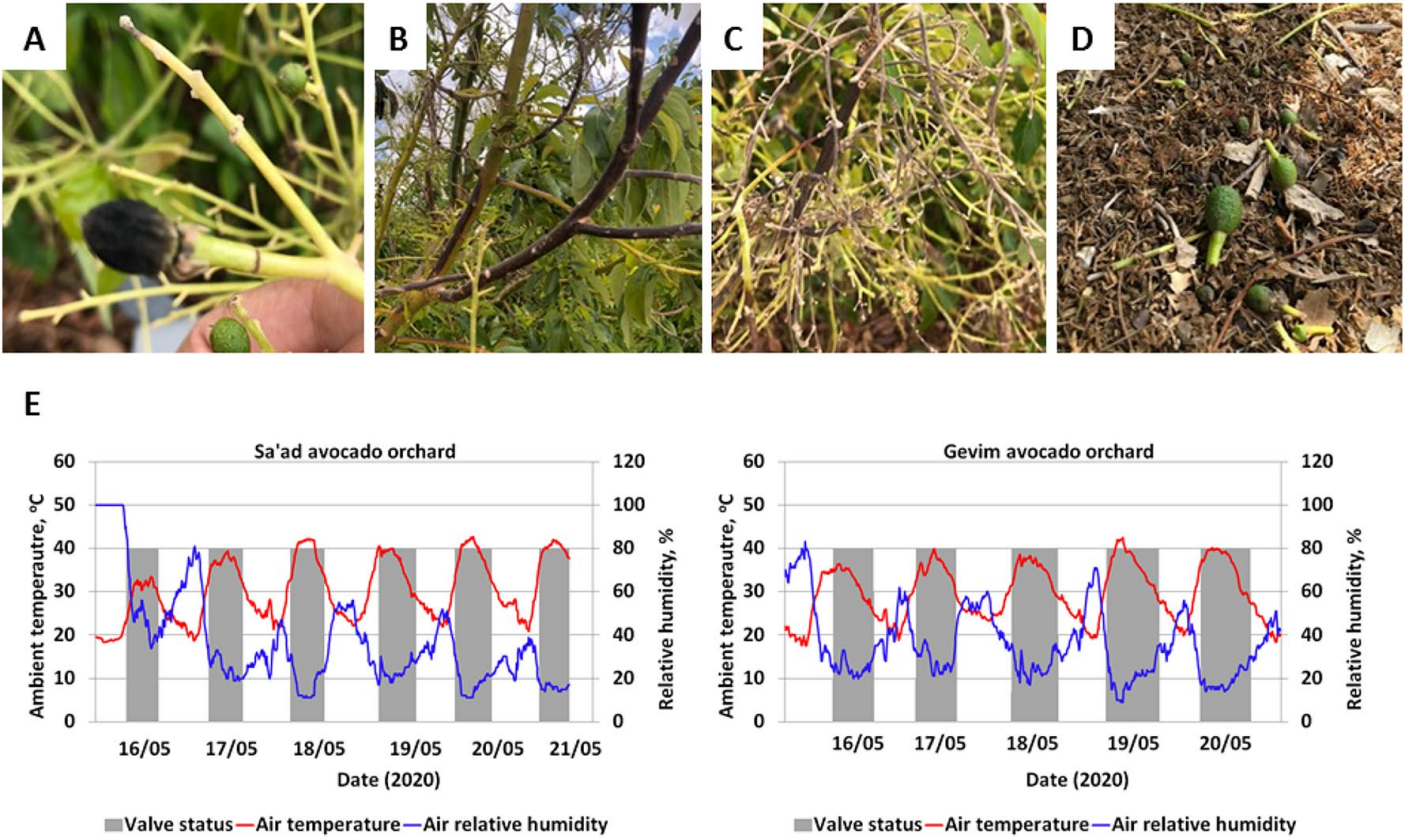
Heatwave damage in avocado orchards. (**A**) Fruitlet burning. (**B**) Branch burning. (**C**) Inflorescence drying. (**D**) Fruitlet dropping. (**E**) The daily air temperature and relative humidity data measured by local meteorological stations during the heatwave period. Valve status: gray rectangle means open valves.

A heatwave began on May 16, 2020, which lasted six consecutive days and caused severe damage to the regional avocado orchards: burned leaves, branches, inflorescences and fruitlets, leaf shedding and fruit dropping (Fig. 2A–D). The ambient conditions were extreme, with the highest temperatures occuring simultaneously with the lowest humidity (Fig. 2E).

**Figure 3:**
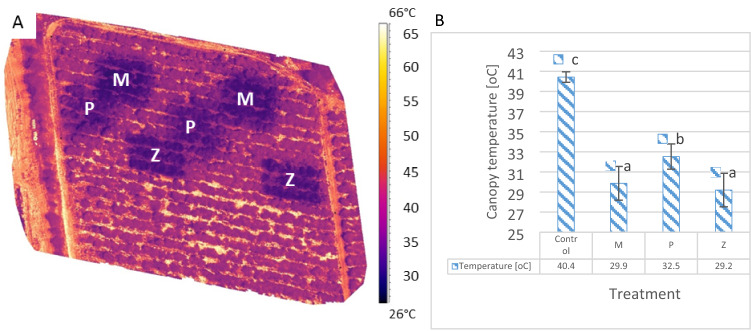
Canopy temperature in the orchards during the heatwave. (**A**) Above-canopy thermal imaging of the Gevim orchard with six experimental plots to which different treatment types were applied. P: sprinklers in pulses (irrigation rate of 17 m^3^/h/ha). M: sprayers (21 m^3^/h/ha). Z: sprinklers (31 m^3^/h/ha). (**B**) Mean values of the tree's canopy temperature (taken from **A**). Different letters represent significant (*p* ≤ 0.05) differences between treatments. Bars are SD values.

The distribution of canopy temperature under the three different cooling treatments in the Gevim avocado orchard is illustrated in Fig. 3. The above-canopy thermal images were acquired during the heatwave, at midday. There were significant differences in foliage temperature between the trees under cooling treatment (dark purple zones) compared to the untreated trees. Image analysis revealed that the control tree canopies were 10 °C warmer than were the treated ones (Fig. 3A, B), and the ground temperature reached up to 64 °C (Fig. 3A). These results indicate that water application at a rate of approximately 20 m^3^/h/ha was sufficient to bring the temperature down to below 30 °C in crown foliage exposed to strong solar radiation.

**Figure 4:**
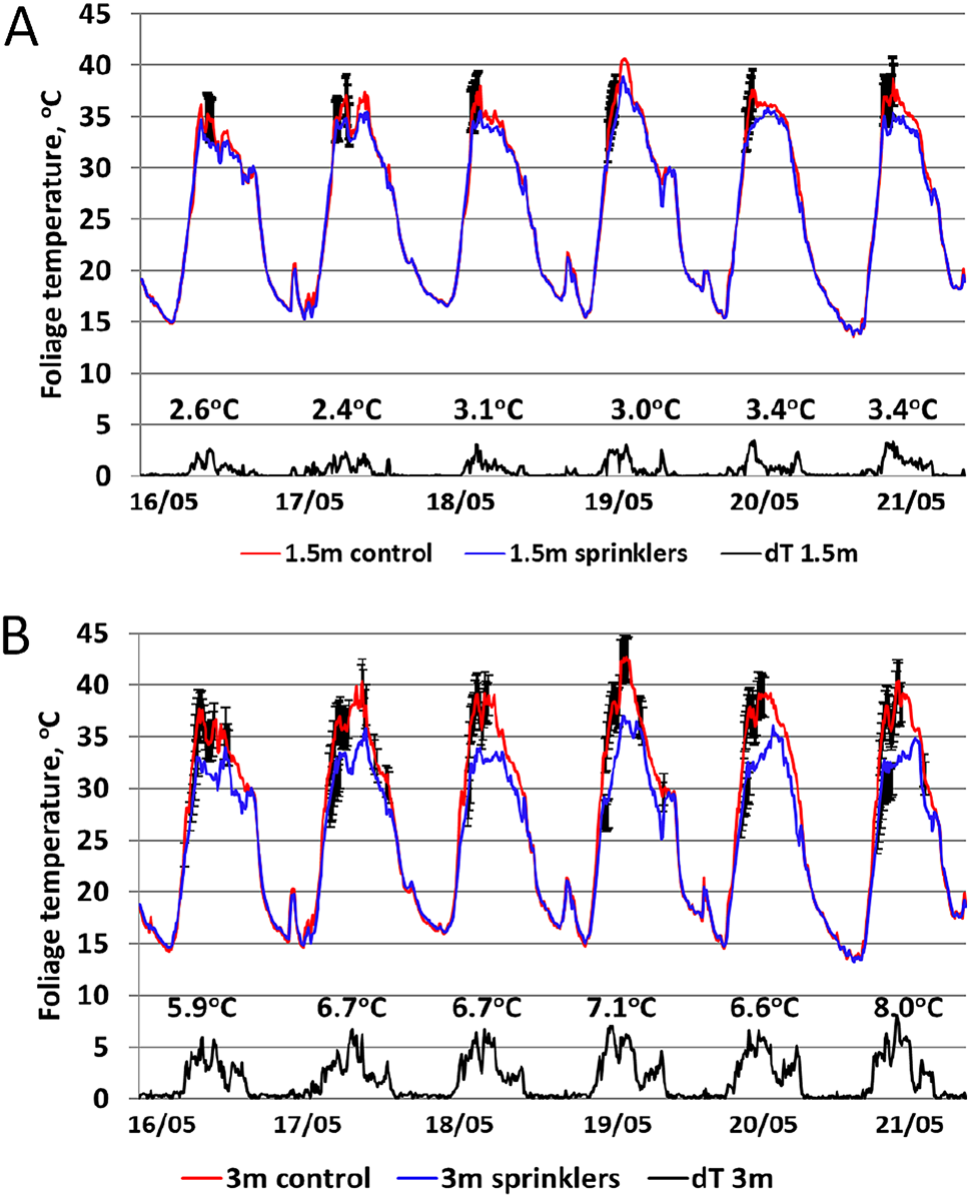
Distribution of foliage temperature inside a tree measured by thermocouples in the Sa'ad orchard: blue line—plot under treatment, red line—control plot, black line—temperature differences between treated and control plots. (**A**) Temperature monitoring at 1.5 m height. (**B**) Temperature monitoring at 3 m height. Values represent the mean of three thermocouple readings at each height. Bars indicate significant differences (P < 0.05) at a given time referring to standart deviation larger than 1 °C up to 3 °C (featuring thermocouples calibration up to ± 0.5 °C). Numbers above the black lines represent the maximal differences of temperatures between treated and control plots.

The foliage temperature distribution inside a tree is illustrated in Fig. 4. The 10-min measurements of inner leaf temperatures measured by thermocouples at heights of approximately 1.5 m and 3 m from the ground were averaged over 2–3 sensor readings at each height. A significant difference in leaf temperature at the 3 m height, of approximately 5 °C, was found between control and treated trees. However, no significant difference was found between the leaf temperatures of the control and treated plots at a height of 1.5 m.

**Figure 5:**
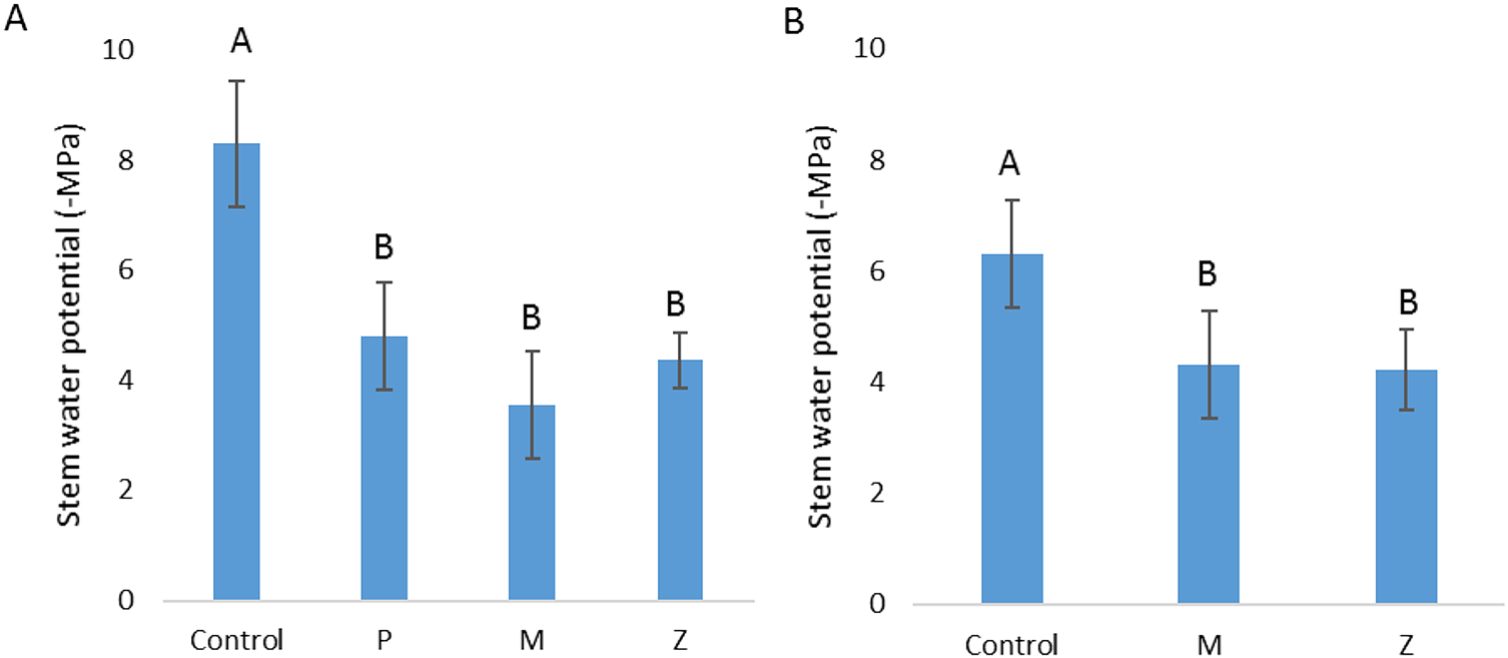
Stem water potential of Hass avocado trees during a heatwave, as affected by several methods of canopy cooling. (**A**) Gevim (BW). (**B**) Sa'ad (FW). P: sprinklers in pulses (irrigation rate of 17 m^3^/h/ha). M: sprayers (21 m^3^/h/ha). Z: sprinklers (31 m^3^/h/ha). Different letters represent significant (*p* ≤ 0.05) differences between treatments. Bars are SD values.

The stem water potential (SWP) was measured during the third day of the heatwave. All three of the cooling methods introduced were found to have a significant, dramatically positive effect over this stress index at both sites (Fig. 5).

**Figure 6:**
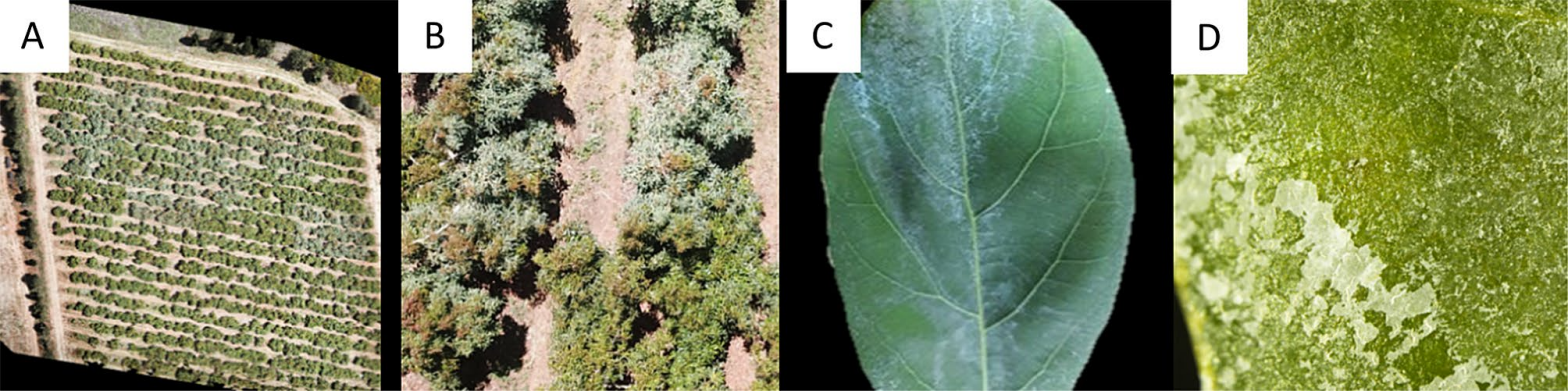
An external white layer on avocado leaves that were treated with brackish water. (**A**,**B**) Drone imaging of the Gevim orchard. (**C**) A treated leaf. (**D**) Mineral crystals on the leaf blade (stereoscope).

The leaves of Gevim's trees, which were treated with BW, exhibited an outer white layer (Fig. 6A–C). The leaves were examined under a stereoscope and the layer seemed to consist of mineral crystals (Fig. 6D).

**Figure 7:**
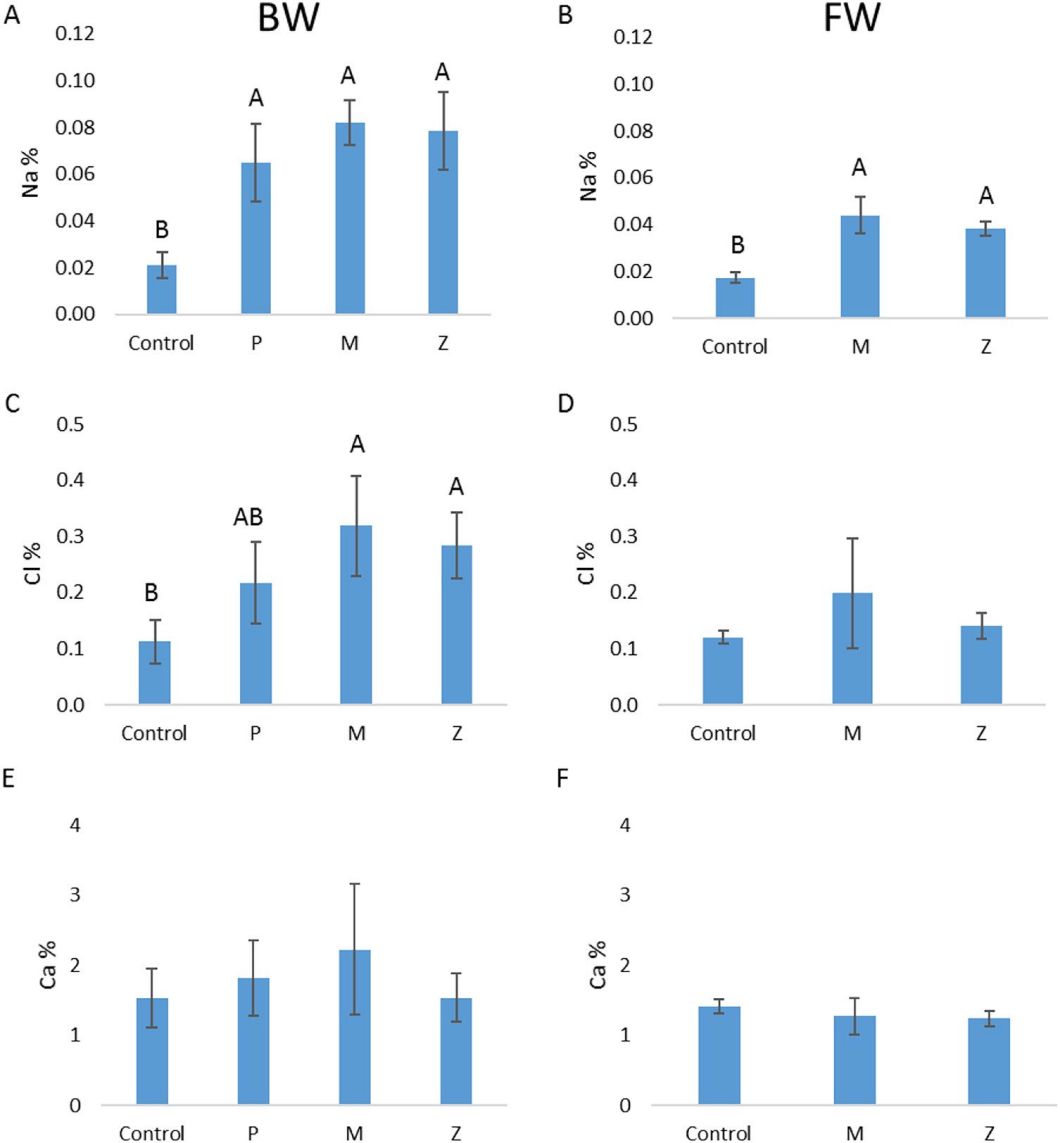
Na, Cl and Ca concentration in Hass avocado leaves, as affected by the methods of canopy cooling studied. (**A**,**C**,**E**) With brackish water (Gevim orchard). (**B**,**D**,**F**) Fresh water (Sa'ad orchard). P: sprinklers in pulses (irrigation rate of 17 m^3^/h/ha). M: sprayers (21 m^3^/h/ha). Z: sprinklers (31 m^3^/h/ha). Different letters represent significant (*p* ≤ 0.05) differences between treatments. Bars are SD values.

Mineral analysis of diagnostic leaves from all the plots revealed significantly higher levels of Na in the treated leaves, both in those using BW and those using FW (Fig. 7A,B). The Cl levels in the leaves treated with BW were significantly higher than were the Cl levels in the leaves of the control trees, except for those that underwent the treatment with pulses of water (Fig. 7C,D). The Ca levels were similar in all the treatments (Fig. 7E,F).

**Figure 8:**
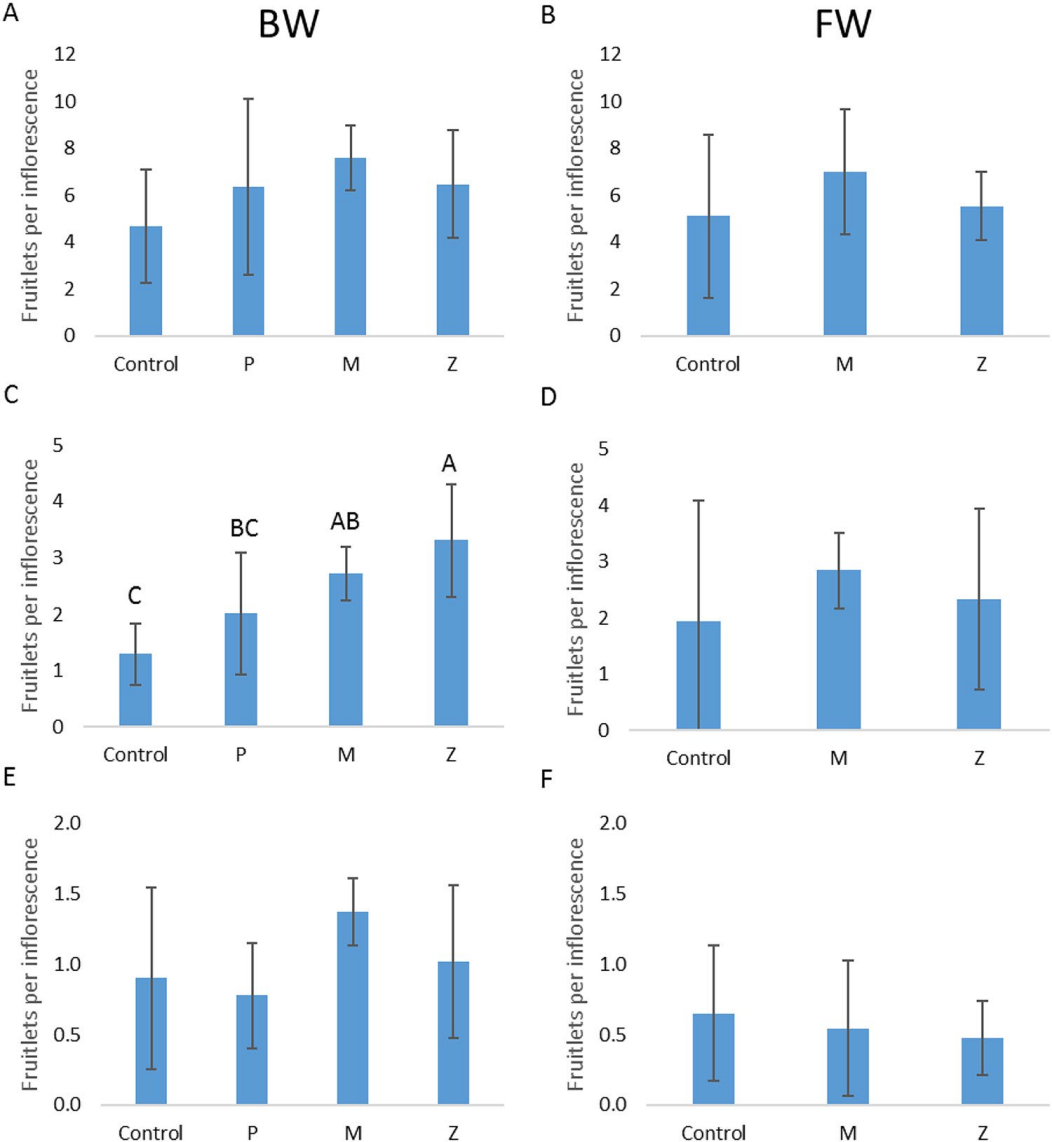
Fruitlets remaining on Hass avocado inflorescences, as affected by several canopy cooling methods. (**A**,**C**,**E**) Brackish water, Gevim orchard. (**B**,**D**,**F**) Fresh water, Sa'ad orchard. (**A**,**B**) 13.5.2020, (**C**,**D**) 27.5.2020, (**E**,**F**) 10.8.2020. P: sprinklers in pulses (irrigation rate of 17 m^3^/h/ha). M: sprayers (21 m^3^/h/ha). Z: sprinklers (31 m^3^/h/ha). Different letters represent significant (*p* ≤ 0.05) differences between treatments. Bars are SD values.

The first fruitlet survey, which was conducted prior to the heatwave, on May 13, 2020, found no significant differences between the treated leaves and the control (Fig. 8A,B). The second survey, which was conducted a week after the heatwave, on May 27, 2020, found that significantly higher levels of fruitlets survived on the trees that were treated by either sprayers or sprinklers, but only in the Gevim orchard (Fig. 8C,D). The third survey, conducted in August, did not reveal any significant effect of the treatments at both sites (Fig. 8E,F).

**Figure 9:**
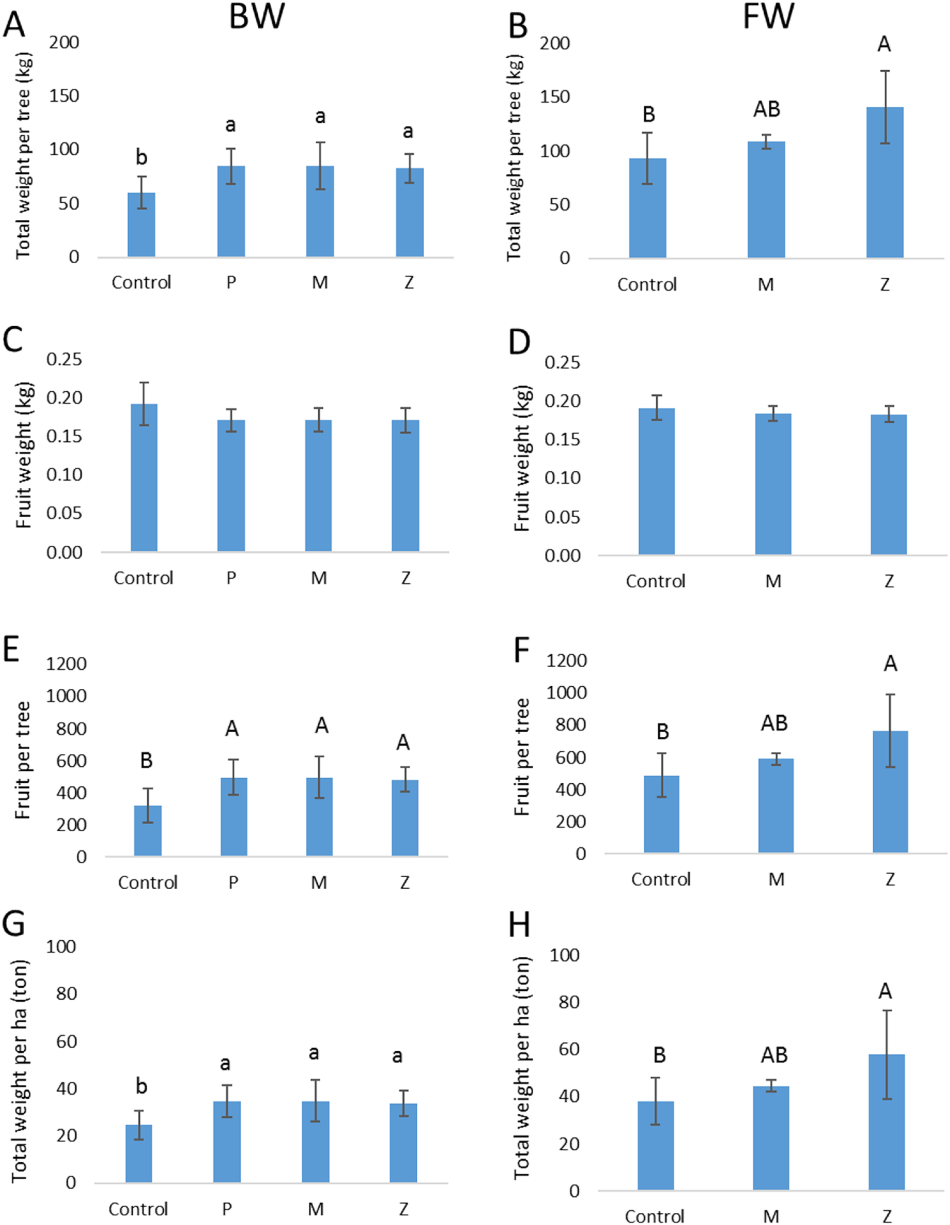
Yield indices in Hass avocado trees, as affected by several methods of canopy cooling. (**A**,**C**,**E**,**G**) Brackish water, Gevim orchard. (**B**,**D**,**F**,**H**) fresh water, Sa'ad orchard. P: sprinklers in pulses (irrigation rate of 17 m^3^/h/ha). M: sprayers (21 m^3^/h/ha). Z: sprinklers (31 m^3^/h/ha). Different capital letters represent significant (*p* ≤ 0.05) differences between treatments. Different lower-case letters represent significant (*p* ≤ 0.07) differences between treatments. Bars are SD values.

The canopy cooling treatments positively affected the total yield (per tree and per hectare) and the number of fruit per tree (Fig. 9). There was no significant influence of the treatments on the fruit weight.

## Discussion

Fruitlet survival is a critical factor in final avocado yield. Despite its abundant flowering, Hass yield is low because of excessive flower and fruit abscission^40^. This abscission has many causes; some are internal due to the nutritional and physiological status of the tree^41^, some are related to the source of the pollen (self vs. cross)^42^ and some are related to external abiotic stresses^43^. Drought and heat are known to be major negative factors in this context, exacerbating immature fruit drop^43^. Nevertheless, 'Hass', which was tested in the current study, is considered to be more tolerant to heat stress than are other cultivars, e.g. 'Fuerte'^44^, so evaporative cooling might be even more beneficial with other cultivars.

Avocado leaves are responsive to changes in VPD; stomatal conductance has been observed to decrease as VPD increases^45,46^. Hence the natural evapotranspiration which naturally cools the tree, is dramatically reduced during heat waves, when the tree needs them the most. The evaporative cooling system provides the water from an external source rather than an internal one, and water evaporates from the entire leaf surface, not only the stomata. Furthermore, the young fruitlets and the pedicel are also cooled by this system, leading to much better cooling than that achieved by natural cooling via evapotranspiration. Coping with the effects of heatwaves on abscission via canopy cooling is based on increasing air humidity and reducing leaf surface temperature by evaporation^47^.

The by-product of enhanced availability of water in the soil is another benefit; it improves the tree's water status and reduces its stress. Water stress during the critical stages of fruit ontogeny, a phenomenon linked to corky lesions developed at the abscission sites, has been attributed to water deficit in trees during fruit development^48^. This fact connects our findings on reduced water stress (as indicated by SWP) to the findings on better survival of fruitlets and increased yield in the trees exposed to evaporative cooling. In our study, the air humidity was similarly increased by all sprinklers, but the water application rate of the pulses was lower, so that less water was needed. Coordinating the irrigation with the cooling system is essential, to avoid water loss and flooding of the root zone, which can be critical for avocado trees^49^.

The thermal images of the tree canopy showed a decrease of approximately 10 °C in the canopy temperature after cooling treatments, although the temperature of the inner leaves decreased by only 5 °C and even less in the lower parts. This difference underscores the high heat stress of the outer leaves—those fully exposed to solar radiation and are the primary photosynthetic organs of the tree. It also reflects the ambient conditions of the fruitlets, which are mainly present at the canopy's outer circumference.

Due to the global shortage of freshwater for irrigation, brackish water is often used in orchards, including avocado^50,51^. Using low-quality water for sprinkling might damage the foliage and decrease the final yield^52^. Avocado is considered one of the most salt-sensitive crops^53^. The accumulation of Cl and Na in leaves following the evaporation of water from the leaf surface leads to severe salinity stress. Apparently, the trichome on the leaf surface^54^ enhances the accumulation of salt crystals on the leaf. However, in our experiment, the salinity exposure by the brackish water that damaged the leaves turned out to have a negligible effect on the tree's health and functioning; the spring vegetative flushes, which are a known characteristic of avocado trees^55^, were healthy and compensated for the whitened leaves (Fig. 6A,B). The white layer quite probably functioned as a temporary mechanical barrier against the radiation, similar to tree trunk whitewashing^56^, and leaf-coating by kaolin spray^57,58^. Nonetheless, the new growth is sensitive to salt accumulation; therefore, the evaporative cooling treatment should last no more than a few weeks when using low-quality water, otherwise substantial damage to the tree foliage might occur.

The average crop yield of Hass avocados is around 10 t ha^−1^^40,59^. Despite the visible damage caused by the heatwave, the yields in the present study were relatively very high in the control trees in both sites, reaching 20–40 t ha^−1^. Nevertheless, several cooling treatments improved the reproductive results, up to almost 60 t ha^−1^, reflecting the better physiological status of the treated trees, as evaluated by SWP measurements during the heatwave. The fruitlet survival surveys did not reveal such differences between the treatment and the control. We assume that the inflorescences marked for the survey did not represent the final yield as they were at lower parts of the tree, while in mature avocado trees, most of the fruit is close to the canopy. As demonstrated in this paper, evaporative cooling is an effective approach to reduce heat stress during heat waves. Another approach that was recently tested is to cover the avocado trees with shading nets^24^. However, this approach is more expensive, and technically more complicated. Moreover, the process and creates several other challenges, like a reduction in pollinators activity.

The cost of the evaporative cooling system is around 12,000 NIS/ha or 3,500 USD/ha. Given that the approximate life span of the system is 10 years, the cost is approximatlly 350 USD per annum. The average price for the grower for 1 kg of avocado in Israel in the recent years is 6 NIS per kg or 1.75 USD, Thus, an increase in yield of 200 kg per ha. should cover the system's cost. In this study, the yield in the treated plot in Gevim increased by 4500 kg/ ha., and in Sa'ad by 9000 kg/ha., using the best evaporative cooling treatment) (Fig. 9). Under those conditions, the establishment and operation of the system is profitable. Of course, profitability depends on the duration, frequency and the severity of heat waves each year. In light of the predicted increased temperatures as the climate change, heat waves are expected to become more frequent and more severe, hence the use of the sytem will be more attractive.

To conclude, our study offers, for the first time, an applicative canopy cooling system, which utilizes the existing irrigation system of the avocado orchard and responds immediately to environmental conditions. This system effectively reduces the physiological stress experienced by the avocado trees during spring heatwaves and improves the final yields when compared with the yields of non-treated trees. The cooling system can be used in different parts of the world, where avocado trees are exposed to heat stress combined with low humidity, such as California, Australia, and South America^21^. We encourage using application methods entailing low water rates so that the irrigation systems can more easily withstand the high flows. To be efficient for the farmer, the canopy cooling system must justify its cost; notably, the same construction can be used to protect the avocado trees from frost damage^60,61^, another natural hazard that might damage this crop.

Climate change cannot be ignored, and heatwaves are already a problem for avocado growers. We propose a viable method to reduce the damaging effect of heatwaves and maintain the profitability of avocado as a crop.

## Data Availability

The datasets used and/or analysed during the current study available from the corresponding author on reasonable request.
